# Therapeutic effect of fecal microbiota transplantation on hyperuricemia mice by improving gut microbiota

**DOI:** 10.3389/fmicb.2025.1599107

**Published:** 2025-08-05

**Authors:** Songjian Yuan, Wenting Jia, Xiaomei Liu, Ruzhen Liu, Man Cao, Yuting Wu, Yuantao Li, Wei Xu, Chuanxing Xiao, Zhenqiang Hong, Bangzhou Zhang

**Affiliations:** ^1^Fujian University of Traditional Chinese Medicine, Fuzhou, China; ^2^Xiamen Treatgut Biotechnology Co. Ltd., Xiamen, China

**Keywords:** fecal microbiota transplantation, hyperuricemia, microbiome, renal injury, gut microbiota

## Abstract

**Objective:**

The primary objective of this study was to assess the impact of fecal microbiota transplantation (FMT) on serum biochemical parameters, renal injury, and gut microbiota in hyperuricemia (HUA) mice.

**Methods:**

Six-week-old male C57BL/6 J mice were given a high-purine diet and potassium oxonate injections to induce HUA, followed by a two-week FMT treatment. Regular body weight checks, serum biochemical analyses, and fecal sampling for 16S rRNA gene sequencing were conducted to evaluate the treatment’s impact on gut microbiota.

**Results:**

The model group showed significant increases in uric acid (UA), creatinine (Cr), blood urea nitrogen (BUN) levels, and increased xanthine oxidase (XOD) activity compared to controls (*p* < 0.05). FMT treatment effectively reduced these levels and XOD activity (*p* < 0.05). At the genus level, specific taxa like *Muribaculaceae* and *Prevotellaceae_UCG-001* were less abundant, while *Blautia* and *Ruminiclostridium_9* were more abundant in the model group. Following FMT, gut microbiota composition returned to near-normal levels, with significant differences from the model group (*p* < 0.05).

**Conclusion:**

This study demonstrates that FMT holds therapeutic potential for HUA mice by reducing UA levels, alleviating renal damage, and restoring gut microbiota balance.

## 1 Introduction

In recent years, there has been a notable rise in the incidence of hyperuricemia (HUA), positioning it as the fourth most prevalent chronic condition, following hyperglycaemia, hyperlipidaemia, and hypertension. Moreover, HUA is recognized as a significant risk factor for the onset of various diseases, such as diabetes, cardiovascular disorders, and atherosclerosis (Wang et al., 2023). Consequently, HUA has become a pressing public health issue (Chen et al., 2016). This condition is characterized by an imbalance in purine metabolism, primarily attributed to either excessive uric acid (UA) production or impaired UA metabolism (Xu et al., 2021). The accumulation of urate crystals can exacerbate the progression of gout and potentially result in chronic nephritis or renal failure. UA is predominantly synthesized through the metabolism of purines in the liver, which can be categorized as either endogenous or exogenous purines. Exogenous purines are typically found in foods rich in protein, fat, purines, and fructose (Kim and Jun, 2022; Skoczyńska et al., 2020). Current pharmacological interventions for HUA generally involve xanthine oxidase (XOD) inhibitors and UA excretion agents, such as allopurinol, febuxostat, and benzbromarone (Mehmood et al., 2019; Yan et al., 2022). However, the prevalence of adverse effects associated with these medications, including liver and kidney damage, nausea, vomiting, dermatitis, and other negative reactions, is notably high (Tien et al., 2022). Moreover, prolonged administration of UA-lowering medications has been linked to a heightened risk of cardiovascular disease in individuals with HUA (Sawada et al., 2023). As a result, there is an urgent need for the development of safer and more effective therapeutic alternatives.

Several studies have identified the kidneys and intestines as the primary organs involved in the elimination of UA. Approximately 70% of UA is excreted via the kidneys, while the remaining 30% is eliminated through the intestines, facilitated by UA transporter proteins in the epithelial cells. Furthermore, the gut microbiota contributes to the degradation of UA within the intestinal tract (Wang et al., 2023). The colonization of gut microbiota in the human intestinal tract establishes a symbiotic relationship with the host, aiding in immune regulation (Su et al., 2022). Modifications in the composition of gut microbiota have been implicated as potential factors contributing to metabolic disorders, particularly HUA. Research has demonstrated a significant reduction in both the abundance and diversity of gut microbiota in HUA mice, along with alterations in the microbial structure compared to the control group (Wei et al., 2022). Specifically, HUA mice exhibited elevated levels of pathogenic and opportunistic bacteria and diminished levels of probiotic bacteria within their gut microbiota (Pan et al., 2020). The gut microbiota plays a crucial role in UA metabolism by facilitating the synthesis of purine-metabolizing enzymes, releasing inflammatory cytokines, and decreasing UA levels.

Fecal microbiota transplantation (FMT) involves the transfer of stool or frozen samples from a healthy donor to a recipient suffering from diseases associated with dysbiosis in the gut microbiota. The primary objective of FMT is to restore microbial balance and enhance microbial diversity in the recipient’s gut by introducing beneficial microorganisms (Shao et al., 2023). Xie et al. demonstrated the efficacy of FMT in reducing UA levels, alleviating gout symptoms, and enhancing intestinal barrier function in patients experiencing acute and recurrent gout (Xie et al., 2022). In another study, Fu et al. reported that modulation of gut microbiota through antibiotic intervention could decrease serum blood urea nitrogen (BUN), creatinine (Cr), XOD, and UA levels, as well as ameliorate renal tubular necrosis in HUA geese on a high-cholesterol and purine (HCP) diet (Fu et al., 2024). Liu et al. observed that although antibiotic treatment reduced UA levels in HUA rats, fecal transplants from HUA rats to recipient rats resulted in increased UA levels in the latter (Liu et al., 2020). The above studies indicate that gut microbiota may play a potential role in the development of HUA, and modulating gut microbiota via FMT may be an effective strategy for managing HUA symptoms.

This study aims to evaluate the efficacy of FMT in treating HUA by modulating the gut microbiota of HUA mice induced by a high-purine diet and potassium oxonate. The gut microbiota composition and diversity in HUA mice were analyzed through 16S rRNA gene sequencing using the Illumina MiSeq platform. Additionally, this study seeks to investigate the mechanisms through which FMT reduces UA levels in HUA mice by examining the role of gut microbiota. The outcomes of this research will contribute to the development of preventive and therapeutic strategies for HUA and provide novel treatment options for affected individuals.

## 2 Materials and methods

### 2.1 Reagents and materials

Potassium oxonate (PO, with a purity of no less than 98%), allopurinol and carboxymethyl cellulose sodium (CMC) were procured from Macklin Biochemical Technology Co., Ltd. (Shanghai, China). Assay kits for UA, XOD, BUN, Cr were supplied by Nanjing JianCheng Bioengineering Institute (Nanjing, China).

### 2.2 Preparation of donor fecal transplant materials

Donor candidates were evaluated through a questionnaire and physical examination to determine their health status, which included assessments of medical history, medication history, family history, and potential risk of infectious diseases. Individuals who smoked or consumed alcohol were excluded from participation, as these factors can disturb the gut microbiota (He et al., 2021). To maintain the integrity of the donated fecal samples, donors were instructed to provide fresh samples in designated containers within a controlled clean room environment. The fecal microbiota was then extracted using an automated fecal microbiota extractor, TG-01 Extn, from Xiamen Treatgut Biotechnology Co., Ltd. (Xiamen, China). The sample was ultimately obtained from the Donor 1101. Fresh fecal specimens were subjected to centrifugation at 5,000 × g for 5 min to isolate a purified microbial fraction. The resultant bacterial cells were then resuspended in normal saline within a homogenizer bag, achieving a bacterial concentration of 1 × 10⁹ CFU/mL. Subsequently, the suspension was aliquoted into sterile tubes and preserved at −80°C for future animal experimentation (Liu et al., 2023; Shaheen et al., 2025). All donor candidates participated voluntarily, signed an informed consent form, and the study was approved by the Ethics Committee of the Third Affiliated Hospital of Fujian University of Traditional Chinese Medicine (Approval No.: 2022-k1-037).

### 2.3 Animals experimental design

In previous experiments, we utilized a model of PO-induced acute HUA in mice and observed minimal pathological damage to the kidney, which aligns with findings from a similar acute HUA mouse model developed by Dhouibi et al. (2021). Consequently, the current study utilized a high-purine diet in conjunction with PO to establish a chronic HUA model in mice. C57BL/6 J mice (5–6 weeks old, weighing 20 ± 2 g) were obtained from Guangdong Pharmachem Biotechnology Co Ltd. (SCXK (GD) 2020-0054). Mice were acclimatized for 1 week prior to experimentation, during which they had ad libitum access to food and water in a well-ventilated room maintained at an ambient temperature of 24°C ± 1°C, with humidity levels at 50% ± 10%, and a 12-h light/dark cycle (lights on from 07: 00 to 20: 00).

**Figure 1:**
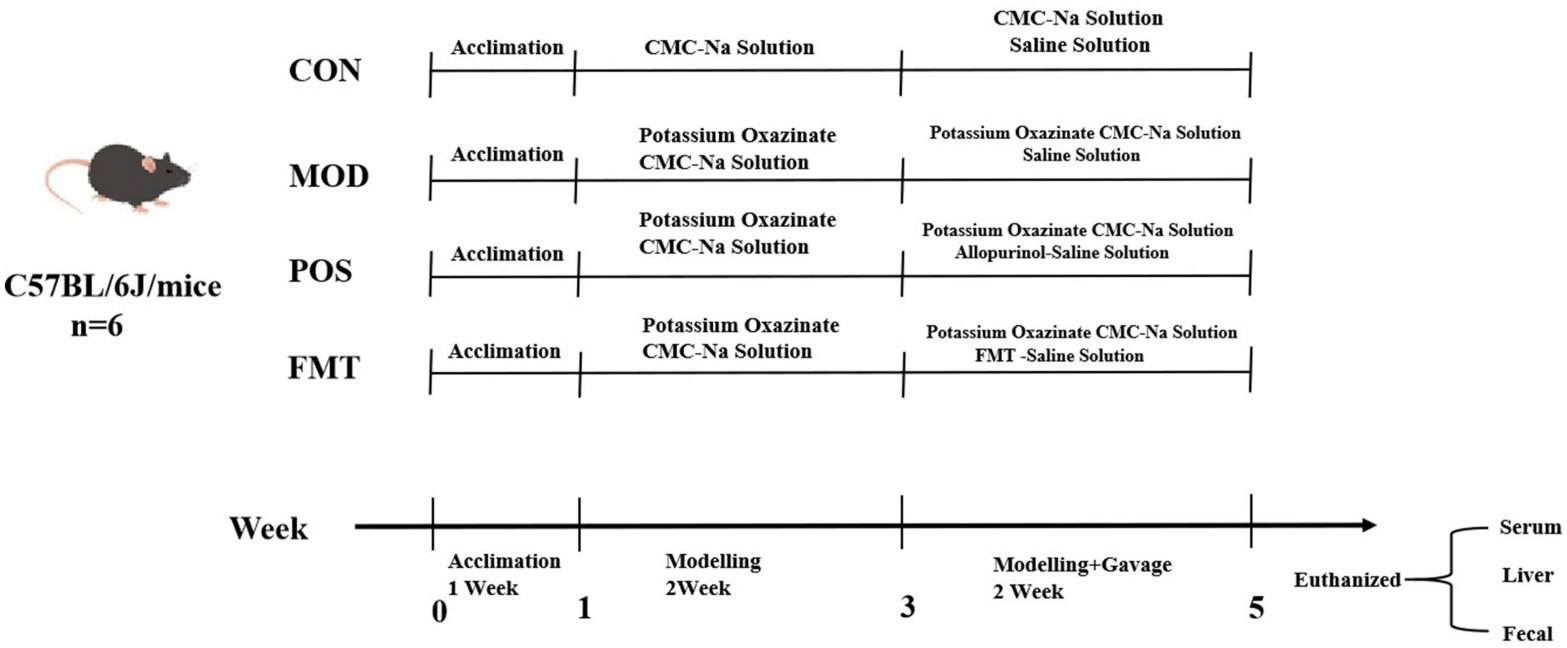
Experimental chart in the treatment of HUA mice.

Thirty-two male C57BL/6 J mice were acclimatized and subsequently divided into four groups based on their initial body weights: control (CON) group, modelling (MOD) group, positive drug (POS) group, and FMT group. The CON group received a standard mouse diet, while the MOD group was provided with a high-purine diet, with all groups having unrestricted access to water. Following a 7-day feeding period, the CON group was administered an intraperitoneal injection of 0.5% CMC-Na solution, while the other groups received intraperitoneal injections of 250 mg/kg/day of PO for 7 days. After 2 weeks of modelling, the CON and MOD groups were given equal volumes of saline, the POS group received 5 mg/kg of allopurinol, and the FMT group was administered 10 μL/g of gut microbiota suspension (with a viable bacterial count of 1 × 10^9^ CFU/mL) for 14 consecutive days. Individual body weights of the mice in each group were recorded every 3 days during the experiment, continuing until the conclusion of drug administration. All animal experiments conducted in this study were approved by the Laboratory Animal Ethics Committee of Fujian University of Traditional Chinese Medicine (Approval No.: FJTCM IACUC 2024201) (Figure 1).

### 2.4 Biochemical analysis

Mice were subjected to fasting and dehydration for 12 h on the 7th and 14th days of modelling, as well as on the 7th and 14th days of treatment. Blood samples were collected from the inner canthus of the mouse eye and placed in a blood collection tubes. Following mixing and centrifugation, the supernatant was transferred to EP tubes for biochemical assays utilizing kits from the Nanjing JianCheng Bioengineering Institute. The parameters measured included UA, BUN, Cr, and XOD activity.

### 2.5 Histological analysis

At the end of the treatment period, mice were euthanized via cervical dislocation, and the kidneys were promptly excised, weighed, and examined for alterations in color, texture, and size of the renal tissues. To observe the detailed histopathological changes, kidneys from the various groups were preserved in a 10% buffered formalin solution, subsequently embedded in paraffin, sectioned into 5–7 μm slices, stained with hematoxylin and eosin (H&E), and analyzed under a light microscope.

### 2.6 DNA extraction

Following 14 days of treatment, fresh stool samples were collected and stored at −80°C for gut microbiota DNA extraction. Genomic DNA from the fecal samples was extracted using the QIAamp Fast DNA Stool Mini kit (Qiagen, CA, United States). The concentration and purity of the isolated DNA were evaluated using spectrophotometry (Multiskan™ GO, Thermo Fisher Scientific, United States). Extracted DNA was purified with a DNA gel purification kit (Qiagen, Germany), and the quality of the DNA extracts was assessed through agarose (1.5%) gel electrophoresis in 1 × Tris-Acetate-EDTA buffer. The purified DNA was stored at −80°C for sequencing.

### 2.7 16S rRNA amplicon sequencing

The variable V4 region of the 16S rRNA gene was amplified using forward primer 515F (5′-GTGCCAGCMGCCGCGGTAA-3′) and reverse primer 806R (5′-GGACTACNVGGGTWTCTAAT-3′) (Cao et al., 2022). Thirty cycles of PCR amplification were conducted (95°C for 3 min, 30 cycles of 95°C for 30 s, 55°C for 30 s, 72°C for 45 s, and finally, 72°C for 10 min) (Sun et al., 2022). Purification was achieved by adding 0.8 times the volume of magnetic beads (Vazyme VAHTS DNA Clean beads) to 25 μL of the PCR product. Sequencing libraries were prepared using Illumina’s TruSeq Nano DNA LT library Prep Kit (Qiu et al., 2022). The quality of the library was assessed on the Qubit@ 2.0 Fluorometer (Thermo Fisher Scientific, Waltham, MA, China) and Agilent Bioanalyzer 2,100 system. Ultimately, the libraries were sequenced on an Illumina MiniSeq.

### 2.8 Bioinformatics and statistical analysis

Raw paired-end reads were assembled using FLASH. Primers were removed with Cutadapt, and the clean tags were generated by removing the lower reads using the Cutadapt (Caporaso et al., 2010). Chimera checking and operational taxonomic unit (OTU) clustering were performed on the clean tags using USEARCH, following the established pipeline. Specifically, all reads were demultiplexed into individual files, clustered at 97% similarity, then the chimera checking was performed using UCHIME in reference mode (Edgar, 2010). Representative sequences were generated, singletons were discarded, and a final OTU table was created. The representative OTU sequences were aligned against the SILVA_132_97_16S database for taxonomic classification via the RDP Classifier.

Subsequent analyses of alpha and beta diversity were conducted using R software. The Vegan package of R software was employed to calculate the alpha diversity index. Principal coordinate analysis (PcoA) and non-metric multidimensional scaling (NMDS) analyses of Bray-Curtis distances were utilized to reflect the beta diversity of the gut microbiota. R software was used to create two-dimensional plots for PCoA and NMDS. Intergroup differences in the gut microbiota composition were analyzed using linear discriminant analysis (LDA) effect sizes (LEfSe), which employed the non-parametric factors Kruskal-Wallis and rank tests to identify features with significant abundance differences between taxa and used LDA to assess the impact of each feature (Segata et al., 2011).

Data processing and statistical analyses were performed using GraphPad Prism version 9.0 and SPSS 27.0 statistical software. Comparisons of multiple sample means were made using the one-way ANOVA test, and pairwise comparisons were made using the LSD test. *p* < 0.05 was considered significant, and *p* < 0.01 was considered highly significant. In addition, all obtained data are expressed as the mean ± standard deviation (SD).

## 3 Results

### 3.1 Effect of FMT on body weight in HUA mice

**Figure 2:**
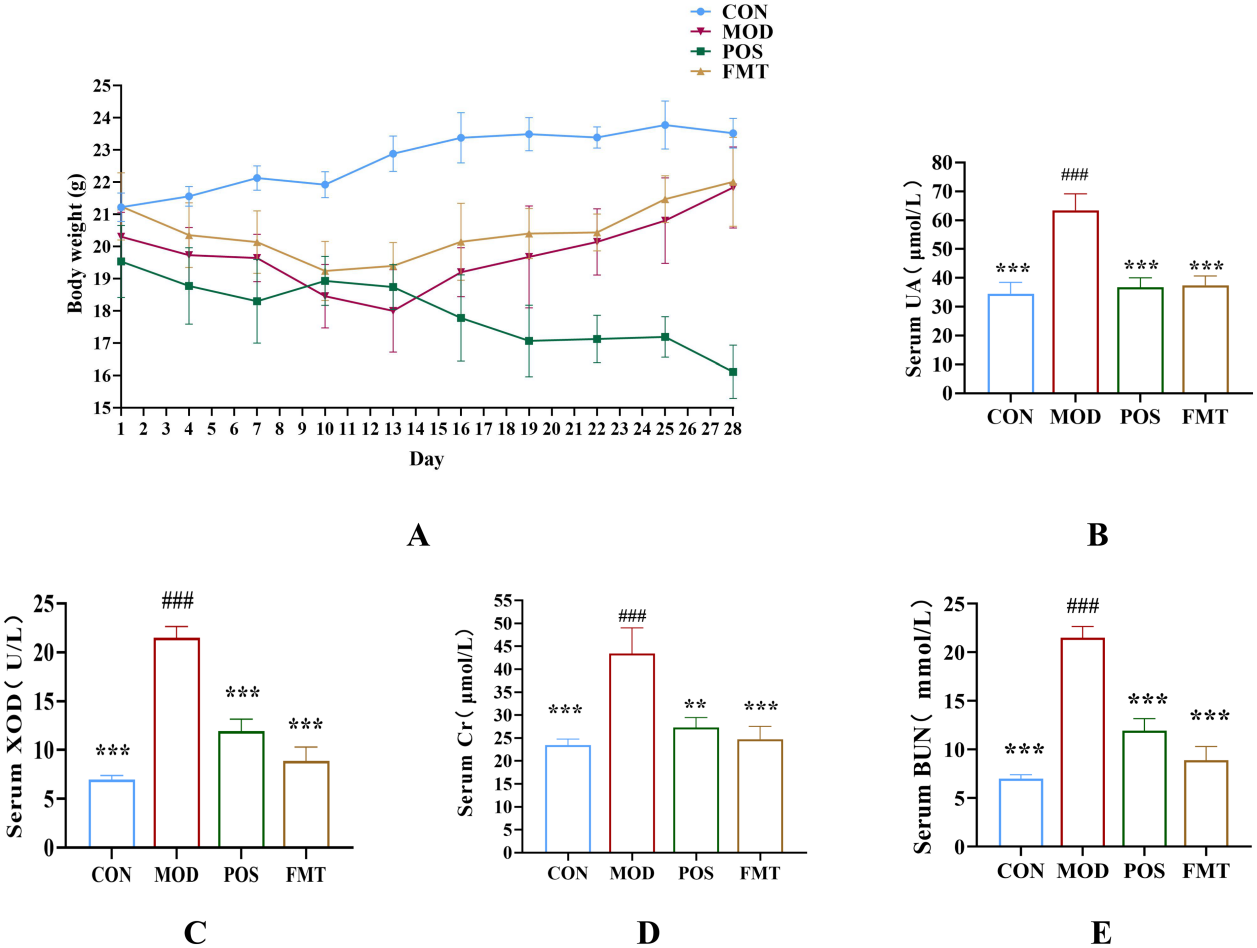
**(A)** Body weight (*n* = 6). **(B)** Concentration of UA from each group. **(C)** Activity of XOD from each group. **(D)** Concentration of creatinine from each group. **(E)** Concentration of BUN from each group. Data are presented as mean ± SD. *** *p* < 0.001, ** *p* < 0.01 and * *p* < 0.05 vs. the MOD group. ^###^ *p* < 0.001 vs. the CON group.

To assess the potential ameliorative effects of FMT on HUA, we established a HUA model in mice through a high-purine diet for the initial 7 days, followed by injections of PO for the next 7 days, culminating in a 14-day modelling period. Commencing on day 15, mice in the CON and MOD groups received saline via gavage, while the POS group was administered the drug allopurinol, and FMT group received a live bacterial preparation via gavage. Following the 14-day modelling period, the body weights of mice in the FMT, POS, and MOD groups were significantly lower than those in the CON group, exhibiting a consistent decline from the onset of modelling (Figure 2A). It is noteworthy that mice receiving FMT gained an average of 3.1 ± 0.6 g during the treatment period, compared to a 1.2 ± 0.4 g loss in the MOD group (*p* < 0.01), indicating a protective effect of FMT against HUA-induced weight loss.

### 3.2 Regulation of serum biochemical indicators in hyperuricemic mice by FMT

UA was a critical biomarker for assessing HUA. Following FMT treatment, the mice exhibited a significant reduction of 26.00 ± 2.21 μmol/L in UA levels when compared to the MOD group (UA: 63.42 (MOD) vs. 37.42 (FMT); *p* < 0.001; Figure 2B). XOD, which catalyzes the conversion of xanthine to UA, demonstrated significantly diminished activity in the FMT group, with a decrease of 6.03 ± 1.38 U/L relative to the MOD group (XOD: 18.38 (MOD) vs. 12.35 (FMT); *p* < 0.001; Figure 2C). Cr and BUN are indicators of kidney damage. In the MOD group, which was subjected to a high-purine diet, these indicators were significantly elevated, indicating varying degrees of renal damage attributable to HUA. Cr and BUN levels in FMT-treated mice decreased significantly compared to the MOD group (Cr: 43.42 (MOD) vs. 24.74 (FMT), BUN: 21.50 (MOD) vs. 8.89 (FMT); *p* < 0.001), as also visualized in Figures 2D,E.

### 3.3 Amelioration of renal injury in HUA mice by FMT

**Figure 3:**
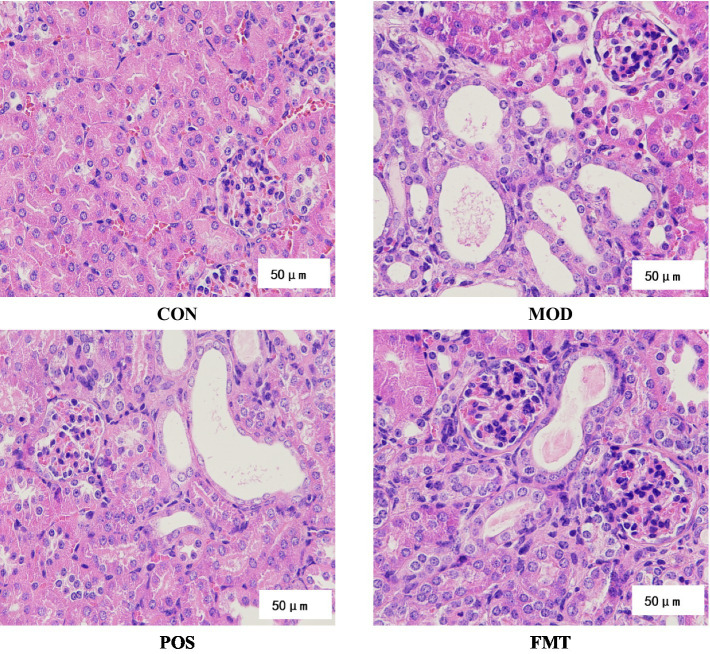
Histopathological analyses of H&E stained kidney sections (×400 magnification) from mice.

The previously described HUA mouse model was established using PO and a high-purine diet. Following FMT treatment, we evaluated its protective effects against kidney injury induced by PO and a high-purine diet in HUA mice. H&E staining (Figure 3) revealed glomerular atrophy and reduced eosin staining intensity of epithelial cytoplasm in HUA mice compared to the CON group. More renal tubules were dilated with flattened epithelium, and eosinophilic proteinaceous fluid was observed within some of the tubular lumens, indicative of a proteinaceous casts in tubular lumens. Additionally, inflammatory infiltrates and necrotic debris were observed in certain renal tubules, signifying severe renal injury induced by PO and a high-purine diet in HUA mice. Importantly, the incidence of glomerular atrophy was diminished in HUA mice treated with FMT, while tubular dilatation was improved, suggesting that FMT treatment can enhance both glomerular and tubular structural integrity.

### 3.4 Treatment effects on gut microbiota diversity of mice by FMT

**Figure 4:**
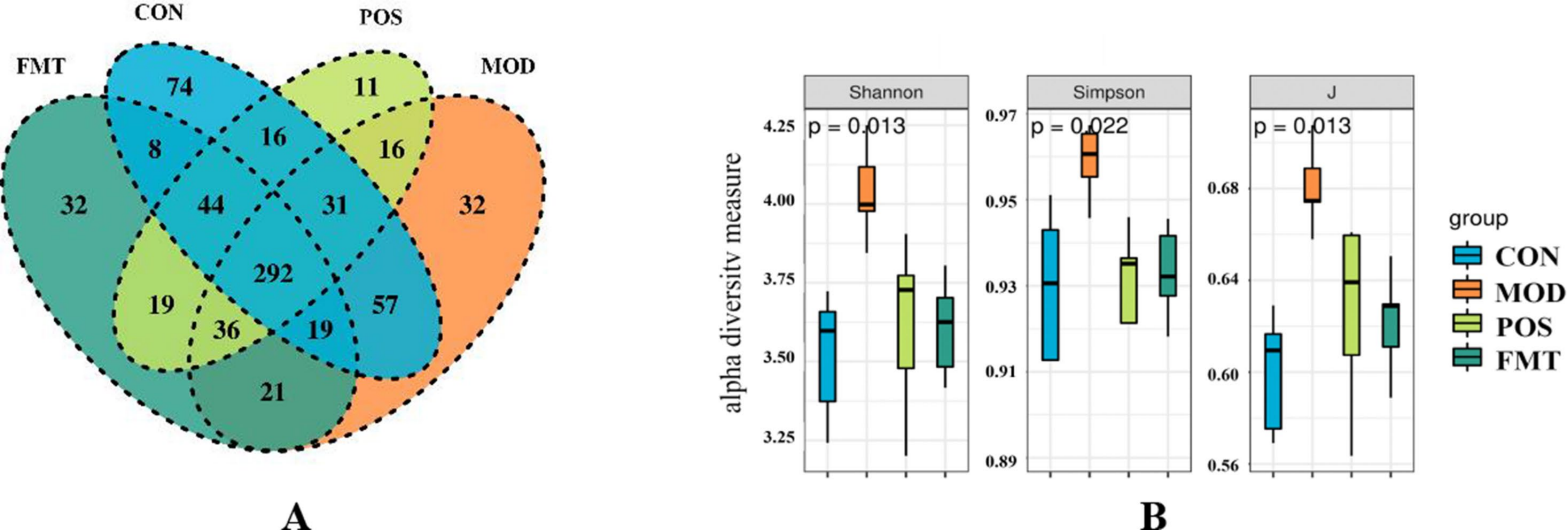
FMT alters the structure of the gut microbiota in hyperuricemic mice. **(A)** Venn diagram; **(B)** Alpha diversity based on the Shannon diversity index, the Simpson diversity index, and the inverse Simpson diversity index (J) in groups of mice.

A Venn diagram was utilized to illustrate the overlap of OTUs among the groups, as well as those unique to each group. As depicted in Figure 4A, community diversity varied across the different mouse groups. Alpha diversity analysis was conducted to assess the microbial community diversity within each sample. The Shannon and Simpson indices, which reflect community richness and evenness, indicated that hyperuricemic mice exhibited higher gut microbiota richness compared to controls, whereas FMT-treated mice did not demonstrate a significant increase in gut flora richness (Figure 4B).

**Figure 5:**
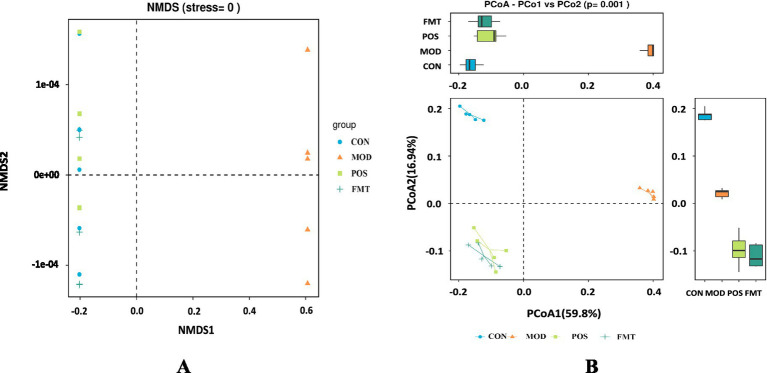
FMT alters the structure of the gut microbiota in hyperuricemic mice. **(A)** Non-metric multidimensional scaling plot (NMDS); **(B)** *β*-diversity based on the Bray-Curtis PCoA method analysis.

The beta diversity of the gut microbiota of mice was assessed using PCoA and NMDS plots. Based on PCoA and NMDS revealed a clear separation among the four groups, indicating distinct differences in community composition (Figures 5A,B). The microbial community structures of the FMT group and the POS groups exhibited significant clustering, which was distinct from that of the MOD group and more closely aligned with the CON group. These results suggest that HUA induces alterations in the structure of the gut microbiota in mice, and that FMT treatment can restore and normalize the community composition of the gut microbiota.

### 3.5 Treatment effects on the taxonomic composition of the gut microbiota by FMT

**Figure 6:**
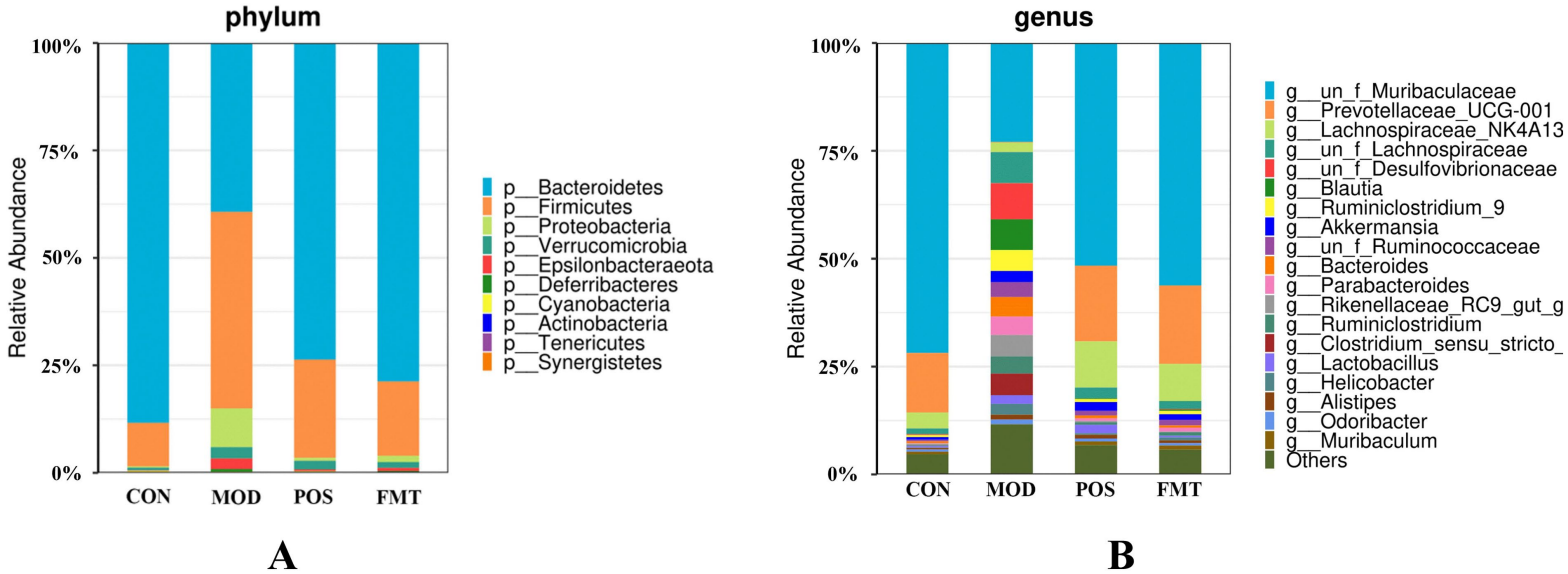
The taxonomic compositions of the gut microbiota in hyperuricemia mice. Relative abundance of the gut microbial community in each group at **(A)** The phylum level. **(B)** The genus level.

**Figure 7:**
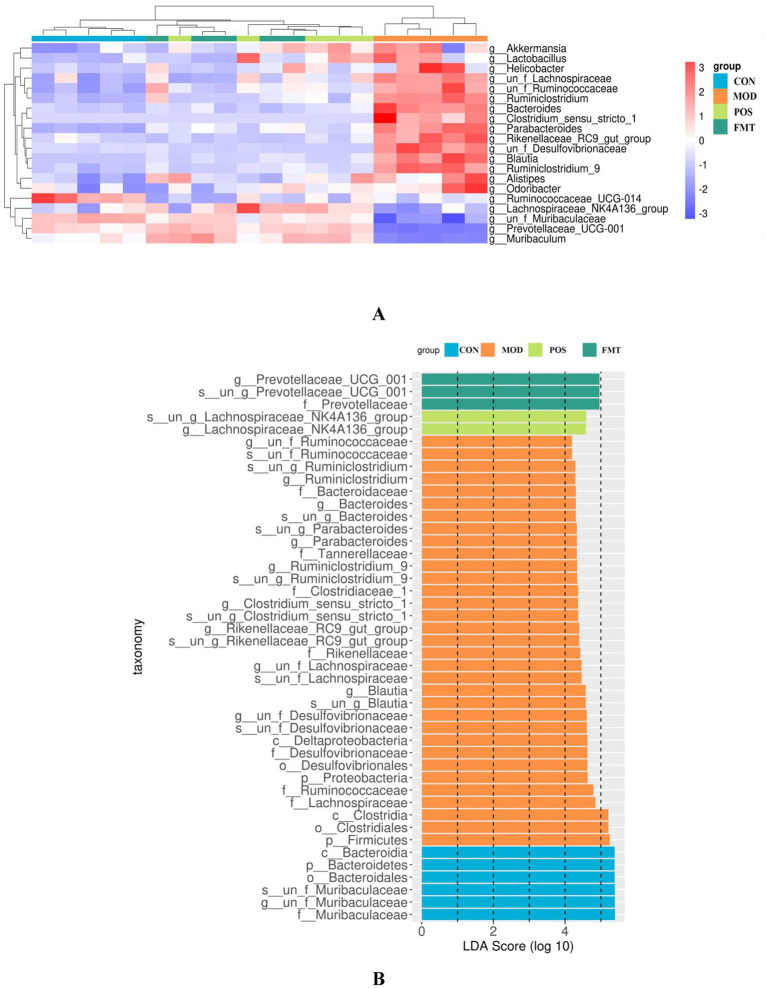
Effect of FMT on the gut microbiota at the genus level in hyperuricemic mice. **(A)** Heat maps of most of the selected differential features at the genus level; **(B)** Differences in gut microbiota composition at various levels derived from LEfSe analyses.

**Figure 8:**
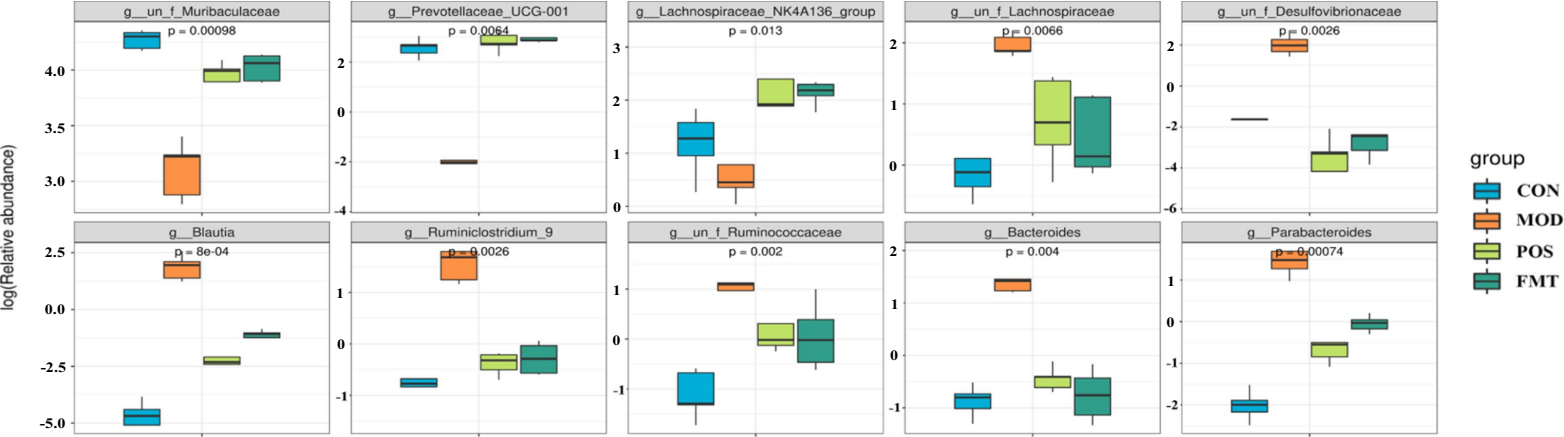
Box plots of genus-level differential species in each group.

At the phylum level, the gut microbiota of mice was predominantly composed of Bacteroidetes and Firmicutes. Notably, the abundance of Firmicutes in the MOD group was significantly elevated, while the abundance of Bacteroidetes was diminished in comparison to the CON group. In the study, the F/B ratio in the gut microbiota of MOD group mice was significantly higher than in the CON group (F/B: 1.16 (MOD) vs. 0.11 (CON); *p* < 0.001; Figure 6). Remarkably, FMT-treated mice showed a significant decrease in the F/B ratio, aligning more closely with the CON group (F/B: 1.16 (MOD) vs. 0.22 (CON); *p* < 0.001). In Akbar Hussain’s experiment on HUA mice using *Limosilactobacillus reuteri*, the F/B ratio of the MOD group showed an upward trend, which reflects microbial imbalance. This implies that metabolic disorders may be impacting urea production and excretion. After administration of *Limosilactobacillus reuteri*, the F/B ratio tended to normalize, and the dysbiosis was adjusted (Hussain et al., 2024). These findings are consistent with our study. To elucidate the changes in bacterial taxa associated with HUA and after FMT treatment, heatmaps and genus-level LEfSe were compared for comparison (Figure 7). Box plots illustrating genus-level difference species across groups revealed that the abundances of *Muribaculaceae*, *Prevotellaceae_UCG-001*, and *Lachnospiraceae_NK4A136* were significantly lower in the gut microbiota of the MOD group compared to the CON group. Conversely, the abundances of *Desulfovibrio*, *Blautia, Ruminiclostridium_9*, *Bacteroides*, and *Parabacteroides* were significantly higher in abundance (Figure 8). The gut microbiota of mice treated with allopurinol and FMT exhibited improvements, aligning more closely with that of the CON group. These results suggest that FMT may rectify the dysbiosis of gut microbiota in mice induced by HUA, potentially serving as one of the mechanisms through which FMT exerts its therapeutic effects on HUA.

## 4 Discussion

The rising incidence of HUA can be attributed to improvements in living standards and changes in dietary habits. Recent statistics indicate that the overall prevalence of HUA in China is approximately 13.3%, with gout prevalence estimated at 1.1% (Tang et al., 2023). The diversification of dietary intake, particularly the excessive consumption of purine-rich foods such as animal offal, legumes, and sardines, contributes to the accumulation of purines within the body (Liu et al., 2021). Furthermore, prolonged fructose consumption has been shown to inhibit renal UA metabolism, leading to elevated UA levels (Caliceti et al., 2017). In addition, meat products contain pro-inflammatory nutrients that may exacerbate HUA (She et al., 2022). Diets characterized by high purine, high fructose, and high-fat content are increasingly prevalent and serve as significant contributors to the development of HUA (Zhou et al., 2022). Consequently, HUA has gradually attracted the attention of the World Health Organization, presenting a global challenge for healthcare systems.

A concerning aspect of HUA management is the potential for serious adverse reactions associated with pharmacological treatments. Allopurinol, for instance, can lead to severe allergic skin reactions, hepatic and renal injuries, and hypersensitivity syndromes such as lethal exfoliative dermatitis. Similarly, febuxostat may induce gastrointestinal symptoms, while benzylbromarone is associated with diarrhoea, nausea, and skin sensitisation (Dubchak and Falasca, 2010). Although UA-lowering drugs are effective in lowering UA, discontinuation of these treatments often results in a rapid increase in UA concentrations (Cheng et al., 2015).

The pathogenesis of HUA is associated with disturbances in purine metabolism and UA excretion. The intestine is an important organ for UA excretion outside the kidney, primarily achieved through the catabolism of gut microbiota and the action of UA transporter proteins (Yin et al., 2022). Liu et al. demonstrated that antibiotic-induced dysbiosis of the gut microbiota alters its composition, elevates UA levels, and modulates purine metabolism in both the host and the gut microbiota (Liu et al., 2023). This suggests a potential link between alterations in the gut microbiota and the development of HUA.

In this study, we investigated the effects of FMT on body weight, modulation of serum biochemical indices, repair of kidney damage, and gut microbiota in hyperuricemic mice. During the modelling phase, the body weights of HUA mice in the MOD group were significantly lower than those in the CON group, indicating that HUA contributes to weight loss. After FMT treatment, the body weight of mice increased significantly compared with the MOD, indicating that FMT can effectively counteract the weight loss caused by HUA. Additionally, compared to the CON group, mice in the MOD group showed significantly higher UA and XOD levels, as well as elevated Cr and BUN levels, which are critical indicators of renal injury. This suggests that the PO-induced HUA model not only increased UA levels in the mice but also caused substantial renal damage. Notably, after 14 days of FMT treatment, there was significant reductions in UA, Cr, and BUN levels, as well as XOD activity, were observed in HUA mice compared to the MOD group, with FMT treatment effects being more pronounced than those observed in the POS group. XOD is an important enzyme that facilitates the synthesis of UA from xanthine, and its inhibition can effectively reduce UA production. Thus, FMT treatment appeared to reduces UA production and promotes its metabolism. H&E staining of renal sections revealed that chronic HUA modelling caused severe kidney damage, including glomerular atrophy, tubular dilatation, and inflammatory cell infiltration. However, FMT treatment significantly ameliorated the pathological structure of glomeruli and tubules in mice. The reduction in Cr and BUN levels further indicated that FMT could ameliorate renal injury associated with HUA. In alignment with our findings, Wang et al. demonstrated that fecal transplantation from guinea pigs into Sprague–Dawley rats, which were induced with 1.5% PO, effectively reduced urinary oxalate excretion, as well as lowered urea, UA, Cr, and BUN levels, suggesting that FMT can mitigate oxalate-mediated renal injury (Wang et al., 2023). H&E staining corroborated that chronic HUA modelling resulted in significant kidney damage, including glomerular atrophy, tubular dilatation and inflammatory cell infiltration. While FMT treatment improved the pathological structure of glomeruli and tubules, underscoring the potential of FMT in addressing HUA-induced renal injury.

To further elucidate the mechanisms underlying the effects of FMT treatment in HUA mice, we conducted 16S rRNA sequencing of the gut microbiota. PCoA revealed that the intestinal microbial diversity in the MOD group was significantly diminished compared to the CON group, exhibiting a distinct clustering pattern. Conversely, the microbiota of the FMT group exhibited greater similarity to that of the CON group. PO-induced HUA disrupted the gut microbiota in mice, with certain bacterial populations playing a pivotal role. At the phylum level, the MOD group demonstrated a significant decrease in the abundance of Bacteroidetes and a significant increase in the abundance of Firmicutes, resulting in a dysregulated F/B ratio. Firmicutes and Bacteroidetes constitute the primary components of the mouse gut microbiota, collectively accounting for approximately 90% of the fecal microbiome (Lee et al., 2021). An imbalance in the F/B ratio has been implicated in the development of metabolic syndrome (Wei et al., 2023). Firmicutes are enriched in genes associated with nutrient transporters and contain numerous carbohydrate-metabolizing enzymes that facilitate the absorption of calories from food, whereas the Bacteroidetes are capable of degrading complex glycans. Thus, Firmicutes are positively correlated with obesity, while Bacteroidetes exhibit a negative correlation, with the F/B ratio is positively correlated with body mass index (BMI) (Baek et al., 2023; Koliada et al., 2017). Research indicates that the abundance of Firmicutes and the F/B ratio are significantly elevated in obese individuals compared to their lean counterparts, while Bacteroidetes abundance is markedly reduced in obese individuals (Cheng et al., 2022). Obesity is closely linked to elevated UA levels, and numerous studies have shown that higher BMI correlates with an increased risk of HUA (Choi et al., 2020; Wang et al., 2022). Zhao et al. identified that stable unhealthy metabolic states and the transitions from metabolically healthy to unhealthy states heighten the risk of HUA among Chinese adults (Zhao and Zhao, 2022). Shailendra Kumar Singh et al. confirmed a robust positive correlation between obesity and UA levels in obese diabetic patients, noting increased XOD activity and active fatty acid synthesis in adipocytes (Singh et al., 2023). The F/B ratio may also serve as a potential biomarker of inflammation in type 2 diabetic patients, with alterations associated with various diseases (Petakh et al., 2023).

At the genus level (Figure 6B), beneficial bacterial populations such as *Muribaculaceae*, *Prevotellaceae_UCG-001*, and *Lachnospiraceae_NK4A136* were significantly diminished in the gut microbiota of the MOD group. *Muribaculaceae*, the dominant bacterial group in the mouse intestine, metabolizes and produces short-chain fatty acids (SCFAs), which are essential for the growth and development of intestinal epithelial cells. Byron J. Smith et al. demonstrated that treatment with acarbose in diabetic mice significantly increased the abundance of *Muribaculaceae*, which primarily produces propionate as a fermentation product, thereby promoting intestinal health in mice (Smith et al., 2021). An increase in the prevalence of *Muribaculaceae* may promote enhanced urinary excretion of UA or inhibit its synthesis, ultimately resulting in a decrease in UA concentrations. In a study by Shimasaki et al., fucoidan oligosaccharide (FOS) was utilized to increase the relative abundance of *Muribaculaceae* in a rat model. This intervention led to a subsequent reduction in serum levels of Cr, UA, and BUN, thereby alleviating renal damage (Shi et al., 2025). Additionally, specific strains within the *Lachnospiraceae* family, such as *Collinsella aerofaciens*, have been found to upregulate genes related to UA metabolism (e.g., *ygeX, ygeW, ygfK*) in uric acid-rich environments. The enzymes encoded by these genes facilitate the degradation of UA into SCFAs. These SCFAs play a role in lowering intestinal pH, which enhances the solubility and excretion of UA, thereby reducing its intestinal levels (Liu et al., 2023). Moreover, *Lachnospiraceae* engage in intricate metabolic interactions with other gut microbiota. Certain species within this family may collaborate with other butyrate-producing bacteria, such as *Roseburia*, to collectively modulate the gut environment, thereby indirectly influencing UA metabolism (Vacca et al., 2020). In the context of harmful strains, the prevalence of *Bacteroides* and *Parabacteroides* was found to be significantly greater in the MOD group compared to the CON group. *Bacteroides*, a conditionally pathogenic bacterium, is known to be overrepresented in the gut microbiota of individuals suffering from HUA, especially those diagnosed with gout (Shao et al., 2017). This overrepresentation of *Bacteroides* has also been documented in various HUA mouse models (Amatjan et al., 2023). Furthermore, our investigation identified a significant increase in the abundance of *Desulfovibrio*, *Blautia*, and *Ruminiclostridium_9* in HUA mice. Numerous studies have established a positive correlation between *Desulfovibrio* and metabolic syndrome phenotypes, with elevated levels observed in patients with coronary artery disease with type 2 diabetes mellitus (Sanchez-Alcoholado et al., 2017; Singh et al., 2023), as well as in women diagnosed with gestational diabetes mellitus (Crusell et al., 2018). Given that HUA is a metabolic disorder characterized by dysregulated purine metabolism, we hypothesize that the heightened presence of *Desulfovibrio* in HUA patients may play a role in the pathogenesis of the condition. Following FMT treatment, the dysbiosis of the gut microbiota in mice was improved, resulting in a reduction of F/B ratio. The abundance of beneficial bacteria taxa, including *Muribaculaceae*, *Prevotellaceae_UCG-001*, and *Lachnospiraceae_NK4A136* increased significantly, while the abundance of harmful bacteria such as *Desulfovibrio*, *Blautia*, and *Ruminiclostridium_9* decreased significantly. The microbial composition shifted towards the composition observed in the CON. Su et al. demonstrated that FMT in patients with type 2 diabetes mellitus led to a reduction in the abundance of *Desulfovibrio* in their fecal samples (Su et al., 2022). Therefore, we speculate that FMT may mitigate the overexpression of harmful bacteria and restores the abundance of beneficial bacteria by enhancing the ecological balance of the gut microbiota in HUA mice, ultimately leading to an improvement in HUA.

In summary, the innovation of this study lies in its exploration of the potential therapeutic role of FMT in the management of HUA, thereby offering novel strategies that extend beyond traditional pharmacological and dietary approaches. FMT not only facilitated weight loss and mitigated renal damage in HUA-affected mice but also significantly influenced the composition and diversity of the gut microbiota, indicating a novel therapeutic target for HUA.

Although FMT has shown promise in alleviating HUA in murine models, its implementation in clinical practice faces numerous obstacles. The complex composition of the gut microbiota, along with individual differences among patients, complicates the ability to forecast treatment outcomes effectively (Yadegar et al., 2024). Various factors, including patients’ genetic profiles, dietary practices, concurrent pharmacological treatments, and existing comorbidities, can significantly impact the effectiveness of FMT (Shao et al., 2023). Furthermore, there exists a potential risk of infection, as donors who appear healthy may harbor pathogens, with serious cases reported among immunocompromised patients (Green et al., 2023). Additionally, further investigation is necessary to assess the relative efficacy of different methods of FMT administration.

Future research should conduct a thorough investigation into the clinical potential of FMT. The integration of multi-omics technologies has the potential to significantly enhance our understanding of the gut microbiota, thereby clarifying the mechanisms that underpin FMT and improving the predictability of treatment outcomes. The development of personalized FMT protocols, along with advancements in donor screening and quality control measures, will be crucial. Additionally, the exploration of innovative administration routes and formulations may broaden the applicability of FMT. These initiatives will facilitate the wider and more effective application of FMT in the treatment of HUA and associated conditions, ultimately providing patients with safer and more effective therapeutic alternatives.

## Data Availability

The original contributions presented in the study are publicly available. This data can be found at: https://www.ncbi.nlm.nih.gov/, PRJNA1135905.
