# Supplementary material for: Therapeutic effect of fecal microbiota transplantation on hyperuricemia mice by improving gut microbiota
Source: Front Microbiol. 2025 Aug 5;16:1599107. doi: 10.3389/fmicb.2025.1599107 (PMC12361192; doi:10.3389/fmicb.2025.1599107)
Supplement: Supplementary file 1 [file Data_Sheet_1.pdf]

Gut Microbiota Analysis of Donor 1101

At the phylum level, the gut microbiota is dominated by Firmicutes and Bacteroidota. At the genus level, *Bacteroides*, *Faecalibacterium*, *Prevotella*, and *Lachnospira* are predominant. After FMT, the abundance of *Prevotellaceae_UCG-001* and *Lachnospiraceae_NK4A136* in mice is higher than in the model group, possibly due to the donor's higher abundance of *Prevotella* and *Lachnospira*.

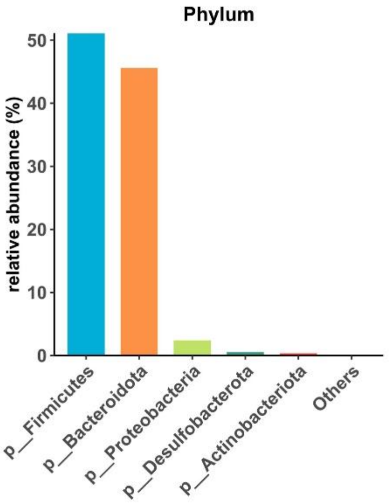

A

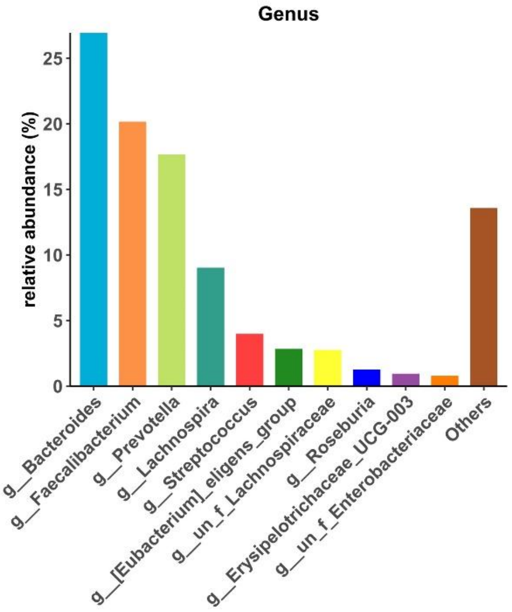

B

(A) The phylum level. (B) The genus level.
